# Enhancing chronic back pain management: A comparative study of ultrasound–MRI fusion guidance for paravertebral nerve block

**DOI:** 10.1515/med-2025-1147

**Published:** 2025-08-19

**Authors:** Shuyao He, Yaona Xu, Bo Li, Ying Shi

**Affiliations:** Department of Radiology, Southwest Hospital, Third Military Medical University (Army Medical University), Chongqing, 400038, China; Department of Digital Medicine, School of Biomedical Engineering and Medical Imaging, Third Military Medical University (Army Medical University), Chongqing, 400038, China; Department of Orthopedics, Beijing Luhe Hospital, Capital Medical University, Beijing, 101199, China; Department of Pain, Shanghai Pudong New Area People’s Hospital, Shanghai, 200000, China

**Keywords:** ultrasound-MRI fusion, paravertebral nerve block, chronic back pain, propensity score matching, clinical effectiveness

## Abstract

**Objective:**

This study examines the efficacy of ultrasound (US)-guided paravertebral nerve block (PVB) with and without MRI fusion for chronic back pain management.

**Methods:**

A retrospective analysis of 20 patients, split into US–MRI fusion-guided (IF group, *n* = 10) and traditional US-guided (U group, *n* = 10) PVB, was conducted. Pain intensity, gabapentin dosage, procedure duration, and treatment efficacy were compared using numerical rating scale (NRS) scores.

**Results:**

An hour after receiving treatment, the IF group showed a marked reduction in NRS scores (2.2 ± 0.9), significantly lower than those observed in the U group (2.5 ± 1.0; *p* < 0.05). Nonetheless, the difference in average NRS scores between the groups was not statistically significant 7 days post-treatment (IF group, 3.5 ± 0.8; U group, 3.4 ± 1.3; *p* > 0.05). The U group reported four instances of transient dizziness and diminished limb muscle strength, lasting between 30 and 90 min, which naturally resolved without intervention. No significant adverse effects were noted in the IF group.

**Conclusions:**

Integrating US with MRI for PVB guidance emerges as a groundbreaking and efficacious strategy in chronic back pain treatment, showcasing significant improvements in safety and initial pain alleviation compared to the conventional use of US guidance alone.

## 1 Introduction

Chronic pain, persisting for over 3 months, significantly diminishes the quality of life for sufferers [1]. The technique of paravertebral nerve block (PVB) guided by ultrasound (US) is a prevalent approach for managing chronic pain, offering prompt pain relief and notable improvement in functional impairments [2]. Nonetheless, this method encounters several obstacles. US imagery is prone to creating shadows over calcified tissues, leading to potential signal disruptions to bone structures [3]. Moreover, in cases involving obese individuals or patients with localized swelling, US’s ability to provide clear imagery is compromised, hindering accurate intraoperative distinction [4]. The advent of US image fusion navigation technology, which facilitates the merging of various images for real-time guidance, enhances contrast and leverages the strengths of different imaging modalities to elevate the precision, efficiency, and safety of both diagnostic and therapeutic procedures. Magnetic resonance imaging (MRI) boasts superior capabilities in delineating spatial and soft tissue contrasts, including fat, muscle, and fascia, without the drawbacks of bone artifact interference, thus offering a more accurate depiction of lesions and adjacent normal anatomical features [5]. Image fusion technology has found extensive applications in diverse medical fields such as tumor physical therapy, stereotactic radiosurgery, image-guided navigation systems, and picture archiving and communication systems. Yet, the integration of US-MRI fusion for directing PVB in chronic pain management remains unexplored.

In light of this, our study introduces and examines the US-MRI fusion-guided PVB technique as a novel intervention for chronic pain treatment. We assessed its feasibility, efficacy, and safety by comparing therapeutic outcomes, adverse reaction rates, and procedural times against those observed with traditional US-guided PVB.

## 2 Methods

### 2.1 Participants and setting

This study received the endorsement of the hospital’s ethics committee, and prior to their inclusion, all participants consented in writing, ensuring their full awareness and agreement to partake. We strictly adhered to confidentiality protocols, safeguarding patient privacy and anonymizing identifiable information throughout the research process.

The investigation incorporated 20 patients who received treatment at the Southwest Hospital of Army Medical University (Third Military Medical University), China, between May 2019 and January 2020, selected based on predefined eligibility criteria. The inclusion parameters were as follows: (1) individuals aged between 20 and 75; (2) patients with a confirmed diagnosis of chronic pain who had not achieved adequate pain relief from standard medical treatments and were candidates for PVB therapy; (3) a numerical rating scale (NRS) score of 6 or higher; and (4) the completion of computed tomography (CT) and MRI prior to the nerve block procedure. The exclusion criteria encompassed (1) significant cardiac, pulmonary, or renal impairments; (2) diabetics with uncontrolled blood sugar levels (fasting glucose > 8 mmol/L, 2 h postprandial glucose > 10 mmol/L); (3) infection at the puncture site or a systemic infection; (4) coagulopathy; (5) any contraindication to nerve block procedures; and (6) contraindications to undergoing MRI scans.

### 2.2 Method of grouping

In this study, 20 patients fulfilling the inclusion criteria were initially considered. To minimize potential biases and confounders, propensity score matching (PSM) was employed to ensure comparability between the experimental (Imaging Fusion; IF) and control (US; U) groups [6,7]. A logistic regression model was used to generate a propensity score for each participant, incorporating the following covariates: gender, age, duration of disease, initial NRS scores, body mass index (BMI), disease type (lumbar disc herniation, radiculopathy, postherpetic neuralgia), and other relevant clinical characteristics.

Patients were matched with the IF group on a one-to-one basis using nearest-neighbor matching with a caliper width of 0.05 standard deviation. After matching, the balance of covariates between the groups was assessed using standardized mean difference (SMD), with SMD <0.1 indicating good balance. As a result, ten patients were successfully matched to form the control group (US; U), maintaining identical compositions in terms of gender and disease type.

### 2.3 Procedures

#### 2.3.1 US-guided PVB

In the control group (US; U), the US was utilized to identify the nerve root, the nerve root artery, and adjacent vessels close to the nerve root’s edge, facilitating the planning of the injection path based on the anatomical structures surrounding the target area. A 50 mm, 23-gauge needle was then carefully inserted from the posterior aspect, with the needle’s tip positioned on the dorsal side of the nerve. This approach was meticulously designed to prevent potential harm to the nearby artery and to maintain a safe distance from the radicular artery. Following precise location verification, a compounded medicinal concoction was administered. This solution comprised 100 mg of lidocaine hydrochloride (2%, produced by Southwest Pharmaceutical), 50 mg of ropivacaine hydrochloride (1%, Naropin^®^, AstraZeneca), and 20 mg of triamcinolone acetonide (40 mg/mL, Transton^®^, Kunming JIDA), all diluted into a 15 mL mixture with 0.9% saline [8].

A total of 6 mL of this blended medicinal mixture was injected into each targeted nerve root. The success of the needle placement was judged by the spread and diffusion of the drug solution around the nerve root, serving as a guide for accurate puncturing. Throughout the injection, the spread of the solution was dynamically monitored, allowing for real-time adjustments to the needle’s position to ensure optimal delivery.

#### 2.3.2 MRI scanning scheme

Utilizing a Siemens Trio 3.0 Tesla scanner, the examination of the lumbosacral vertebrae spanned from the upper margin of the L1 vertebra down to the lower boundary of S5, employing both the basic and body coils for scanning. Subsequent to this, coronal views of the lumbosacral nerves were captured utilizing a 3D-SPACE (Sampling Perfection with Application optimized Contrasts using different flip angle Evolutions) sequence. This sequence was strategically positioned based on sagittal scans, extending from the frontal edge of the vertebral body to the spinous processes of the lumbar spine. The 3D-SPACE sequence was characterized by specific technical parameters, including a field of view (FOV) of 420 mm × 420 mm, a matrix size of 184 × 184, a repetition time (TR) of 3,800 ms, an echo time (TE) of 293 ms, an inversion time (TI) of 170 ms, a slice thickness of 1.0 mm with no interval between slices, employing a single slab with a total of 80 slices, a number of signal averages (NSA) set to 2, the phase encoding direction being right to left (RL), and a signal-to-noise ratio (SNR) fixed at 1.00. The acquired 3D imaging data were then processed on a local post-processing workstation, enabling enhancements through maximum intensity projection (MIP) and multi-planar reconstruction (MPR) techniques.

#### 2.3.3 US-MRI fusion-guided PVB

**Figure 1:**
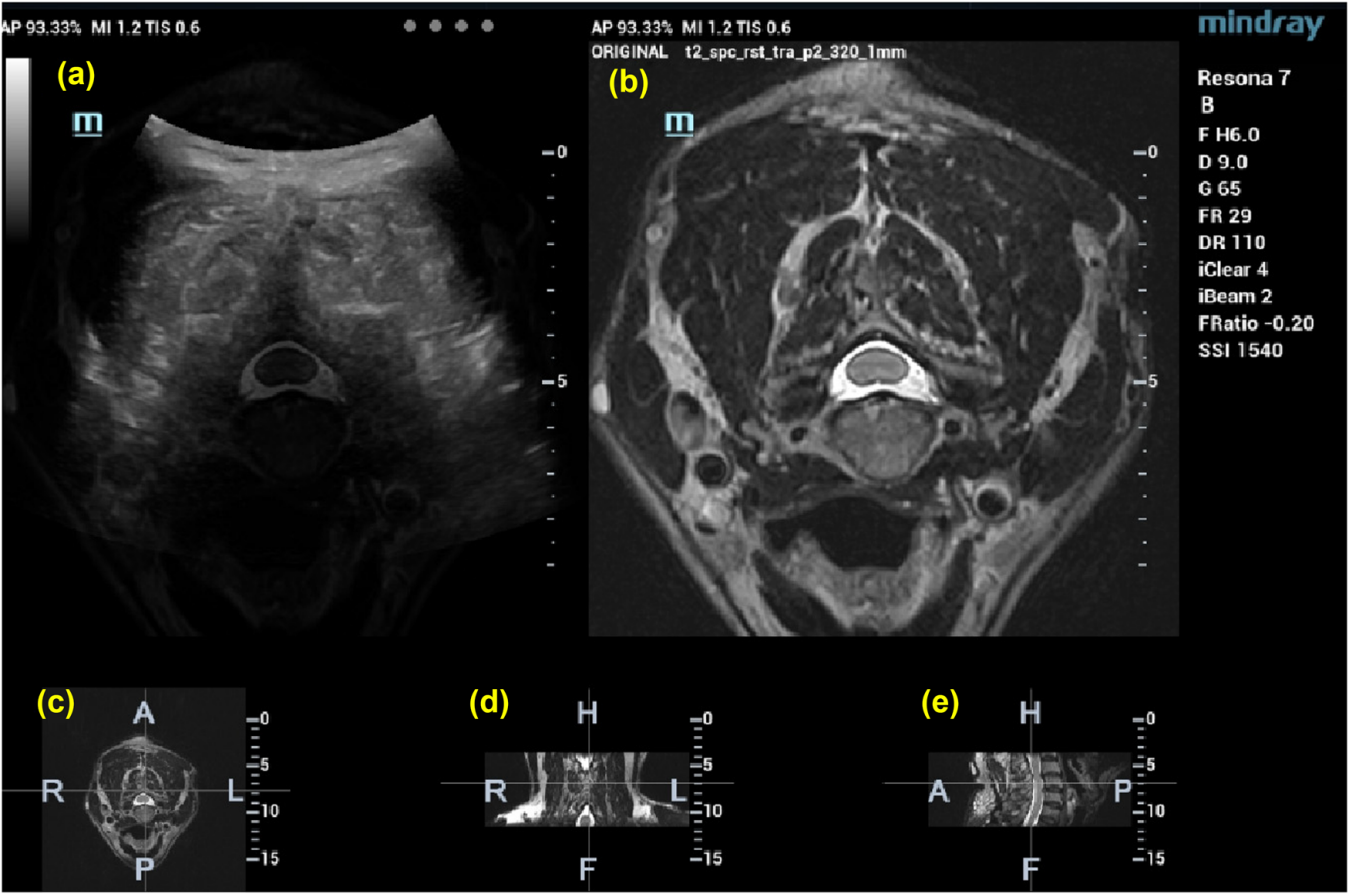
Images of cervical vertebrae after US-MRI fusion: (a) US-MRI fusion images, (b) MRI images, (c) cross section, (d) coronal section, and (e) sagittal section.

**Figure 2:**
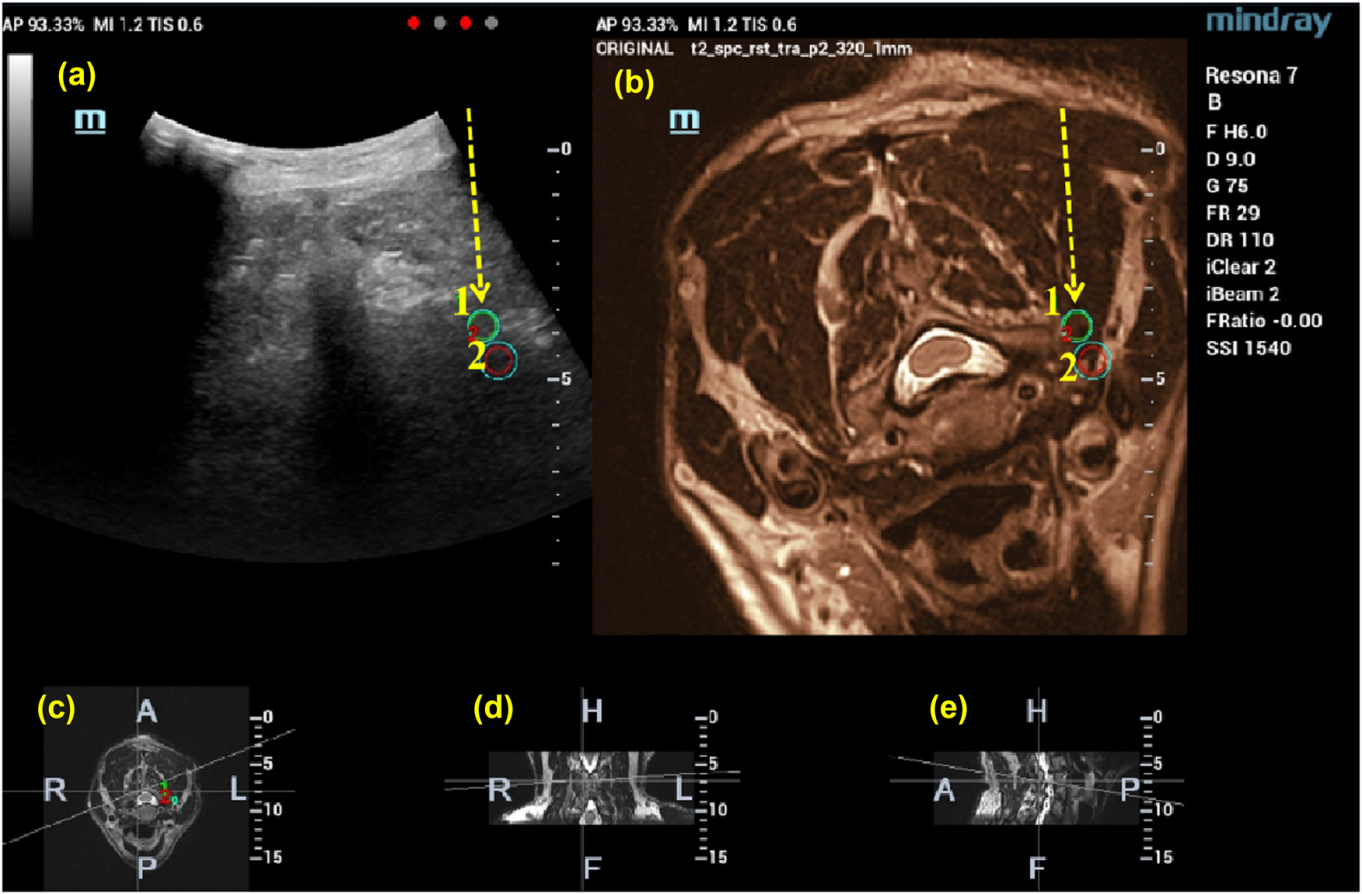
The therapeutic target is marked on the MRI image, and the location of the target is also displayed on the fused image at the same time (1, 2). Then, the puncture path is designed according to the MRI and US images (shown by the yellow arrow). (a) US-MRI fusion images, with green circles indicating the target puncture points, (b)MRI images, (c) cross section, (d) coronal section, and (e) sagittal section.

**Figure 3:**
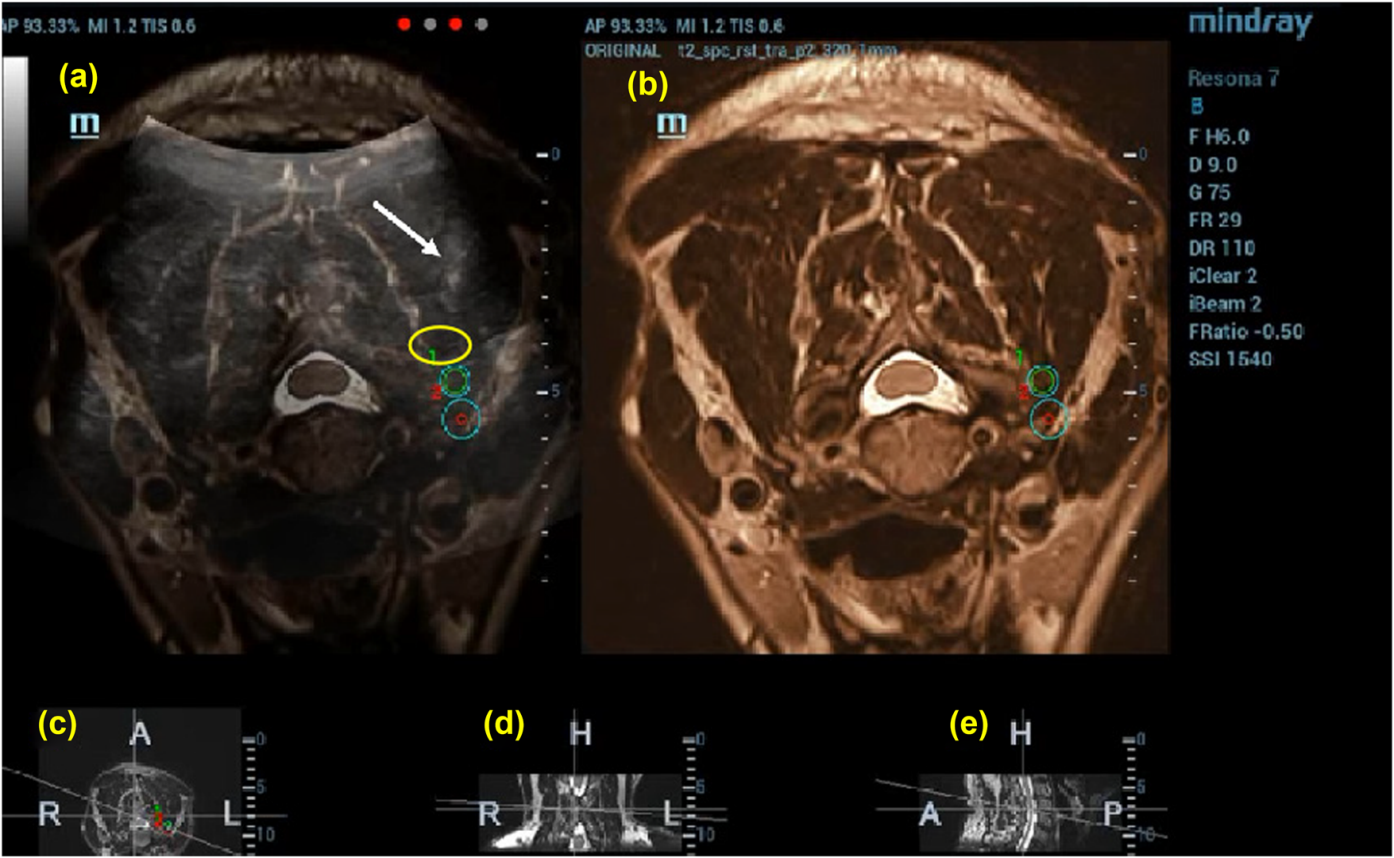
Puncture to preset target 1 guided by US (50%)-MRI (50%) image. The drug solution is injected after the puncture is guided by US (50%)-nuclear magnetic resonance (50%) image; the white arrow indicates the location of the puncture needle and the yellow circle indicates the solutions diffusion range. (a) US-MRI fusion images, with green circles indicating the target puncture points, (b)MRI images, (c) cross section, (d) coronal section, and (e) sagittal section.

In the Imaging Fusion (IF) group, the process began with acquiring preoperative MRI scans, which were then integrated into the control unit of a multi-image fusion interventional navigation system. The procedural steps were as follows:

1. High-resolution MRI scans were conducted using a 3.0T MRI system (MAGNETOM Skyra 3.0T, Siemens, Amberg, Germany), with scanning parameters tailored to the specific characteristics of the target tissues, resulting in 1.5 mm-thick MRI slices.
2. The MRI data, formatted in DICOM (Digital Imaging and Communications in Medicine), were uploaded to the core system of a multi-image fusion interventional navigation device (Mindray, model RE7, China).
3. The connection of the magnetic transmitter facilitated the activation of the image fusion mode. Image registration was achieved by aligning the MRI images with the ultrasonic probe’s imagery and the adjustable screen, utilizing surface markers, bone landmarks, nerves, and other anatomical features as references for accurate matching (Figure 1).
4. The intended therapeutic target was pinpointed on the MRI images, guiding the formulation of a puncture trajectory based on the anatomical structures adjacent to the target (Figure 2).
5. Following the predetermined puncture path, the insertion was performed under US guidance, with the needle directed towards the target, aided by a puncture stand for precision.
6. Once the needle was accurately positioned, ensuring no blood, fluid, or gas was aspirated, the therapeutic medication was administered. The spread of the therapeutic agent was monitored in real-time via US, observing its diffusion around the target area (Figure 3).

#### 2.3.4 Demographic and clinical characteristics

Baseline demographic and clinical characteristics were collected, including age, sex, disease categories, site of pain, and disease course.

### 2.4 Measures

#### 2.4.1 Pain intensity

The degree of pain was quantified using the NRS, where patients rated the most intense pain experienced within the 24 h preceding the evaluation. Pain levels were divided into four categories: no pain (0), mild pain (1–3), moderate pain (4–6), and severe pain (≥7) [9].

#### 2.4.2 Treatment effect

The effectiveness of the pain management strategy was determined by the NRS Weighted Value (NRS-WV), with outcomes categorized into four levels: cured (NRS-WV ≥ 75%), significant improvement (50% ≤ NRS-WV < 75%), improved (25% ≤ NRS-WV < 50%), and no effect (NRS-WV < 25%).

#### 2.4.3 Operation time

The duration of the procedure was meticulously recorded, encompassing the time taken for target localization, the search for the target point via US, and the administration of the drug.

#### 2.4.4 Average daily dose of gabapentin

The mean daily dosage of gabapentin was calculated for the week preceding and following the treatment, providing insights into the medication usage patterns.

### 2.5 Statistical analysis

Preliminary studies investigating the impact of US-guided paravertebral block (US-guided PVB) on NRS pain scores in patients with chronic pain demonstrated a median reduction of 4.6 points. Based on these findings, a cohort of 20 patients was determined to be sufficient to achieve 90% statistical power to detect a significant difference in pain scores pre- and post-treatment, utilizing a two-sided significance level of 0.05 and assuming a standard deviation (SD) of 2 in pain scores. Additionally, a power analysis conducted using PASS 15 software, focusing on the operation time data presented in Table 4, indicated that the current study possesses a statistical power of 100%. Consequently, the selected sample size is adequate to identify significant differences with high confidence, ensuring the validity and robustness of our findings. Experimental data are expressed as mean  ±  standard deviation (mean  ±  SD). To evaluate the significance of differences in post-treatment pain scores, efficacy rates of pain treatment, operation durations, and average daily doses of gabapentin between the two study groups, independent samples *t*-tests were employed. Following PSM, covariate balance between groups was assessed using the SMD of <0.1 considered indicative of negligible differences and effective matching. A *P*-value < 0.05 was regarded as statistically significant.

## 3 Results

### 3.1 Baseline data

The initial demographic and clinical profiles of participants are detailed in Table 1. Prior to treatment initiation, there was no significant disparity in the duration of the illness (4.3 ± 2.8 weeks for the US [U] group vs 4.4 ± 2.8 weeks for the Imaging Fusion [IF] group) or in the average NRS pain scores (7.1 ± 0.9 for the U group vs 7.0 ± 1.0 for the IF group), establishing a comparable baseline between the two cohorts (*p* > 0.05 for both indicators).

**Table 1:** Baseline data after PSM

Group	Sex (male/female)	Age (yr)	Disease course (week)	NRS	BMI (kg/m²)	Disease type	SMD
U (*n* = 10)	6/4	46.8 ± 16.5	4.3 ± 2.8	7.1 ± 0.9	24.5 ± 3.2	Lumbar disc herniation (4), radiculopathy (3), and postherpetic neuralgia (3)	0.05
IF (*n* = 10)	6/4	46.6 ± 12.69	4.4 ± 2.8	7.0 ± 1.0	24.7 ± 3.1	Lumbar disc herniation (4), radiculopathy (3), and postherpetic neuralgia (3)	0.04

Values are presented as mean ± standard deviation. U group: control group; IF group: experimental group; NRS: numerical rating scale for pain intensity; BMI: body mass index; and SMD: standardized mean difference.

### 3.2 NRS scores

When comparing NRS pain scores post-treatment, the IF group demonstrated a notably lower average score (2.2 ± 0.9) 1 h after treatment compared to the U group (2.5 ± 1.0; *p* < 0.05) as presented in Table 2. However, there was no significant difference in the average NRS scores 7 days post-treatment between the groups (3.5 ± 0.8 for the IF group vs 3.4 ± 1.3 for the U group; *p* > 0.05), indicating a convergence in pain relief outcomes over time.

**Table 2:** Comparison of NRS 1 h and 7 days after treatment

Group	1 h NRS	7 days NRS
U	2.5 ± 1.0*	3.5 ± 0.8
IF	2.2 ± 0.9*	3.4 ± 1.3

Values are mean ± SD, U group, IF group, numerical rating scale (NRS), *p < 0.05: comparison of U and IF groups.

### 3.3 Total effective rate of pain treatment

The comparison of the average NRS weighted value (NRS-WV) revealed no significant difference between the groups either 1 h (0.64 ± 0.16 for the U group vs 0.68 ± 0.14 for the IF group) or 7 days after treatment (0.51 ± 0.10 for the U group vs 0.50 ± 0.19 for the IF group; *p* > 0.05 for both), as shown in Table 3.

**Table 3:** Comparison of NRS-WV

Group	1 h NRS-WV	7 days NRS-WV
U	0.6373 ± 0.1646	0.50616 ± 0.1036
IF	0.6778 ± 0.1445	0.50219 ± 0.1902

Values are mean ± SD, U group, IF group, numerical rating scale weighted value (NRS-WV).

### 3.4 Operation time

Operation durations, summarized in Table 4, showed a significant difference; the U group experienced a considerably shorter procedure time (754 ± 89.05 s) compared to the IF group (1516 ± 134.39 s; *p* < 0.05), highlighting the time efficiency of using US guidance alone.

**Table 4:** Comparison of operation time

Group	Operation time (s)
U	754 ± 89.05145*
IF	1516 ± 134.3859*

Values are mean ± SD, control group (U group), experimental group (IF group), *p < 0.05: comparison of U and IF groups.

### 3.5 Average daily dosage of gabapentin

Table 5 outlines the gabapentin dosages before and after treatment. The 7-day pre-treatment average daily dosages were similar between the U group (1.38 ± 0.20) and the IF group (1.41 ± 0.23). Post-treatment, there was no significant difference in dosage changes between the groups (0.99 ± 0.41 for the U group vs 0.96 ± 0.67 for the IF group; all *p* > 0.05), indicating comparable medication adjustments.

**Table 5:** Comparison of average daily dosage of gabapentin

Group	Average daily dose of gabapentin within 7 days before treatment (g)	Average daily dose of gabapentin within 7 days after treatment (g)
U	1.38 ± 0.1990	0.99 ± 0.4069
IF	1.41 ± 0.2343	0.96 ± 0.6681

Values are mean ± SD, control group (U group), and experimental group (IF group).

### 3.6 Incidence of adverse events

Post-treatment, four patients in the U group experienced transient symptoms of dizziness and decreased limb muscle strength, lasting between 30 and 90 min, which resolved spontaneously. The IF group reported no significant adverse effects, underscoring a potential advantage in safety with the use of imaging fusion guidance.

## 4 Discussion

PVB is heralded as a principal therapeutic approach for managing chronic pain [10]. US-guided PVB has been extensively validated in preceding research and stands on par with CT-guided techniques in terms of efficacy [11,12,13,14]. This method offers the advantage of continuous, real-time procedural guidance devoid of the risks associated with radiation exposure.

Nevertheless, the application of US guidance is not without its challenges. As highlighted by Chumnanvej et al., the technique’s visual field is significantly restricted due to the high acoustic impedance presented by bone, rendering US less effective for clear delineation of spinal anatomy in the lumbar area [15]. Consequently, certain minimally invasive pain management interventions cannot rely solely on US guidance and necessitate the adjunctive use of digital subtraction angiography or CT for comprehensive guidance. Furthermore, the aging process affects the echo intensity of muscle tissue under US, markedly increasing the whiteness and brightness of US images in older patients, thereby impairing tissue differentiation [16,17]. The calcification associated with aged, degenerated discs further complicates their distinction from bone via US [12]. Additionally, the presence of adipose tissue can diminish the clarity of US images, affecting both the precision and success rate of interventions and potentially leading to adverse outcomes [18]. These limitations underscore the “bottleneck” encountered in the advancement of pure US guidance technology, signaling a pressing need for innovative solutions to enhance its utility in clinical practice.

The utilization of MRI for the diagnosis and guidance in treating soft tissue and spinal bony lesions has been established as a feasible, effective, and safe method [19,20]. Key benefits of MRI include the absence of ionizing radiation, its capacity for high tissue contrast, and the flexibility of acquiring images in multiple planes [21]. Research conducted by Sequeiros et al. and Himes et al. has demonstrated the successful application of MRI guidance in treating spinal lesions, yielding promising outcomes [22,23]. However, reliance solely on MRI guidance introduces certain limitations, notably extending the duration of procedures due to the necessity for specialized puncture needles and the inability to provide real-time and dynamic guidance during interventions. Additionally, the bulky nature of MRI equipment and the complexity of its operation necessitate dedicated spaces and specialized technicians, significantly constraining the broader adoption of this technology.

In this forward-looking study, we scrutinized the disparities in both efficacy and safety of PVB when guided by US-MRI fusion versus traditional US guidance alone. The data revealed that the NRS scores for both groups significantly decreased 1 h and 7 days after treatment compared to pre-treatment scores. Notably, the NRS scores for patients who underwent PVB with US-MRI fusion guidance were significantly lower than those for patients receiving US-guided PVB 1 h post-injection, indicating that US-MRI fusion guidance surpasses pure US guidance in achieving short-term pain relief. This immediate analgesic effect is primarily attributable to the local anesthetic action in the injected solution, with the enhanced accuracy in needle placement near the nerve root under image fusion guidance likely contributing to the lower pain scores.

However, no significant difference was observed in NRS scores or the average daily consumption of gabapentin between the two groups after 7 days, as shown in Tables 1 and 5. The sustained therapeutic impact of the nerve block over this period predominantly stems from the long-lasting anti-inflammatory and analgesic effects facilitated by the gradual release and penetration of triamcinolone acetonide into the surrounding tissues. This similarity in long-term therapeutic outcomes suggests that despite the initial advantages of US-MRI fusion in terms of precision and immediate pain relief, both methods demonstrate comparable efficacy in managing chronic pain over a week, underscoring the potential of US-MRI fusion guidance as a valuable tool for enhancing the immediate effectiveness of PVB procedures without compromising long-term treatment outcomes.

In the cohort receiving US-guided PVB (US-guided PVB), four patients experienced adverse reactions post-treatment, including dizziness and a decline in lower limb muscle strength. These complications may be attributed to inadequate imaging quality of the paraspinal tissues, which compromised the efficacy of US guidance. The reduced imaging clarity could lead to inadvertent damage to surrounding blood vessels and tissues during multiple needle adjustments. Gofeld et al. [12] noted that the visibility of lumbar nerve roots and blood vessels depends on the depth and echogenicity of adjacent tissues, suggesting that nerves or blood vessels not clearly visualized might be inadvertently pierced by the needle, a finding that aligns with our observations. Additionally, during US-guided procedures, the nerve root may not be well visualized, increasing the risk of the needle tip damaging the nerve sheath or local anaesthetic infiltrating the subneural space, which could enhance motor nerve blockade and lead to reduced limb muscle strength.

Additionally, to further mitigate potential confounding factors between groups, we employed PSM. The matching process included covariates such as gender, age, disease duration, initial NRS scores, BMI, and disease type. The matching results indicated that after PSM, there were no significant differences in any covariates between the groups (SMD < 0.1, *p* > 0.05), ensuring comparability. This matching procedure enhances the credibility of our findings by reducing the impact of potential confounders on the study outcomes.

Remarkably, none of the patients who underwent US-MRI fusion-guided PVB reported adverse reactions akin to those seen with US guidance alone. This outcome underscores the capability of MRI to effectively bridge the gaps in US’s imaging of vascular and neural structures, thereby enhancing the treatment’s efficiency. The integration of MRI with US guidance evidently aids practitioners in more precisely navigating the needle, significantly reducing the likelihood of inadvertently puncturing blood vessels or nerves and, consequently, the occurrence of adverse events following the procedure. This suggests a considerable improvement in safety profiles for patients undergoing PVB with US-MRI fusion guidance, highlighting the method’s potential to refine procedural accuracy and minimize risk.

The method we have introduced, while promising, is not without its drawbacks. Initially, the process of target positioning and the duration of procedures under US-MRI fusion image guidance are considerably lengthier than those guided by US alone, which could potentially impact patient comfort and procedure throughput negatively. Additionally, the small sample size of our study limits the robustness and generalizability of our findings, necessitating further validation of the proposed method’s efficacy and safety within a larger patient cohort. Moreover, the brief follow-up period employed in our study precludes any assessment of the long-term comparative effectiveness of the two guidance techniques. To address these limitations, future research efforts will focus on expanding the participant base and extending the follow-up duration.

The integration of US and MRI technologies in our study has shown significant promise in enhancing the precision and safety of nerve block procedures. The combination leverages the real-time imaging capability of US with the superior tissue contrast of MRI, providing more accurate needle placement and reducing the risk of inadvertent damage to surrounding structures. This is particularly beneficial in complex anatomical areas where US alone may not provide sufficient visualization.

Moreover, the lower incidence of adverse reactions in the fusion imaging group suggests that the enhanced precision directly translates into improved patient safety. The ability to visualize and avoid critical structures more effectively reduces the likelihood of complications such as nerve damage and vascular injury. While the initial costs of US-MRI fusion technology are higher, the potential for reducing the incidence of adverse events and improving clinical outcomes may offer cost savings in the long term. Fewer complications mean less need for additional treatments and shorter recovery times, which could offset the higher upfront investment.

Our study specifically observed that at 1 h post-treatment, the pain scores in the US-MRI fusion group were superior to those in the US-guided group. This early indication of efficacy suggests that the fusion technique provides immediate benefits in pain management. Additionally, the incidence of adverse reactions was lower in the fusion group, reflecting better safety outcomes.

Furthermore, although this study achieved between-group comparability in baseline characteristics through PSM, we also considered other potential confounding factors, such as BMI. BMI may influence patients’ perception of pain as well as their response to PVB treatment. Therefore, future research should further investigate the potential impact of variables such as BMI on the efficacy of PVB therapy to ensure the comprehensiveness and accuracy of the findings.

Future research targeting areas where US guidance alone is challenging, such as the dorsal root ganglia and lumbar intervertebral discs, is expected to further demonstrate the advantages of fusion imaging technology. This ongoing work aims to expand the clinical applications of US-MRI fusion, potentially establishing it as a standard of care in more complex cases.
